# Use of Kaplan-Meier and Cox regressions in the distribution of length of stay in animal shelters for pre-specified calendar periods: Definition, computation, and examples of dog length of stay in orange county California

**DOI:** 10.1371/journal.pone.0342102

**Published:** 2026-01-30

**Authors:** Michael Loizos Mavrovouniotis

**Affiliations:** Social Compassion and SCIL, Laguna Beach, California, United States of America; University of Illinois Urbana-Champaign College of Veterinary Medicine, UNITED STATES OF AMERICA

## Abstract

Computations of length of stay in animal shelters rely on fixed animal cohorts. This is appropriate for research studies that pre-select cohorts, but it is problematic for operational assessments of animal shelters in fixed calendar periods or for comparisons among periods or shelters. Considering only the length of stay of animals whose stay ended within the study period leads to misinterpretation. The use of the Kaplan-Meier and Cox proportional hazards methods with left-truncation and right-censoring is proposed to correctly account for all animals present in the shelter for any fraction of a study period, including those that were present at the beginning and those that remain in care at the end of the period. Examples of dog length of stay in Orange County Animal Care in California show that this computation method corrects the misleading view of historically used calculations of length of stay. Statistically significant changes in length of stay are observed in 8 out of 23 quarterly periods. In a comparison of length of stay before and after the COVID-19 pandemic, the observed significant change in length of stay cannot be explained by variations in sizes and ages of incoming dogs and may be connected to operational policies that restricted visitor access. The proposed approach enables timely tracking of length of stay, accurate comparisons, and assessment of shelter practices and resource needs.

## 1 Introduction

The concept of Length of Stay (LOS) in animal shelters is critical for managing shelter capacity, improving animal welfare, and assessing and improving shelter performance. For an individual animal, the length of stay measures how much time the animal is under the shelter’s care, from intake to outcome [1]. Intake and outcome data from animal shelters for animal cohorts of interest can be summarized into LOS distributions and averages. Currently, however, shelters compute LOS mean only for animals that exit the shelter, distorting calendar-period dynamics because they ignore residents present at the end of the period. This article seeks to remedy this deficiency.

For a fixed number of intakes, lower LOS means fewer animals in care each day, which in turn translates to improved welfare [2]. Staffing needs depend directly on the number of animals in care [3]. Accelerating outcomes and reducing LOS, when feasible, creates care capacity for those animals who require longer stays [4]. Decision-making and animal movement should therefore optimize LOS [1].

We can draw an analogy with (human) hospital length of stay, which has long been used by hospitals to improve patient care, reduce overall costs, and allocate resources according to patient needs [5]. Hospital LOS affects resource requirements, costs, and capacity planning [6]. It can serve as an indicator of hospital unit efficiency and patient flow [5] across hospital environments or over time. Like hospitals, animal shelters seek the best appropriate outcomes for individuals in their care but cannot disregard LOS in assessing their policy choices and resource allocation. The most widely-used shelter metric is the Live Release Rate [7], which captures the ratio of live to total outcomes regardless of LOS. But LOS cannot be ignored in shelter operations, as it governs capacity and resource availability (which may in turn impact a shelter’s ability to maintain or increase its live release rate). Unnecessary days in the shelter increase crowding, compounding shelter-acquired illness and behavior decline [8,9]. Reducing average LOS improves staff and resource efficiency while ensuring animals do not spend needless nights in the shelter. Shelters are accordingly advised to remove barriers and delays to obtain faster outcomes [10].

Multiple studies have examined the effects of factors such as size, age, breed, behavior, health, fostering, and labeling on LOS [11–20]. A large multivariate study [21] determined that breed, purebred status, size, sex, neuter status on arrival, age, coat color, veterinary history, and the shelter all affect LOS. The study used Kaplan-Meier (KM) fits and Cox proportional hazards regressions, statistical methods common in veterinary epidemiology [22] and employed in this article. Other studies using Cox regressions considered the impact of animal or shelter factors on dog LOS [23], time to adoption [24], the relative rates of adoption and euthanasia [25], and cat LOS [26]. These studies involve pre-defined cohorts of animals, e.g., all animals entering the shelters of interest in a specific period. We do not review them in detail, because we have a different focus, namely the examination and comparison of LOS in pre-defined calendar periods.

For on-going assessment of shelter operations and performance, tracking LOS changes, specifically in annual and monthly periods, is recommended [8]. Average LOS can be computed over the animals with outcomes in each period to detect long-term trends [27]. When shelters use this approach for shorter periods, however, they have a distorted view of shelter operations, because they disregard the fact that some animals’ stay overlaps only partially with the period under consideration. When, during some period, long residents accumulate in the shelter, they are not reflected in the LOS, concealing the problem. When long residents leave in a later period, the LOS metric of this departure period is perversely penalized.

One software vendor’s description of available reports [28] specifies that Total Length of Stay (Report 272) includes animals that have an outcome (“outgoing status”). An aggregation of data from approximately 1,000 shelters by a different vendor [29,30] illustrates this point: At the onset of the COVID-19 pandemic in March–April 2020, pages 18–19 [30] show a sharp reduction in the dog population (because total outcomes exceed intakes) with a surprising sharp increase in LOS. In that period, more long residents exited the shelter. Thus, LOS calculations based on animals exiting each month show an increase, as if animals are moving more slowly through the shelter, when the opposite is the case. Similar information is present in the vendor dashboard [31]. A follow-up discussion with the panelists states [32]: “LOS (or more accurately, average LOS) is a calculation that occurs once an animal has left the shelter, and measures how long it took before that dog or cat had a live or non-live outcome”.

An ASPCA webinar [33] shows calculation of LOS (page 6) based solely on animals with outcomes. This is driven, in part, by the subcategorization of LOS by outcome type, such as median LOS for cat adoption (page 61) or mean dog LOS from intake to adoption (page 65) [33]. Organizations recognize the consequences of LOS on resident count, as can be seen in [33] pages 73–76; and [34] page 28. But a calculation using only animals with outcomes undermines this relationship, as the computed LOS is lower when long residents do not leave the shelter; and appears, instead, elevated when these long residents leave. Thus, methods currently in use in animal sheltering produce biased LOS estimates for fixed calendar periods.

The primary purpose of this study is the presentation of a methodology that remedies the flaws of LOS metrics currently in use in animal shelters. An example of LOS trends for dogs in the Orange County, California, animal shelter serves to illustrate the methodology. In addressing the discrepancy inherent in shelter LOS computations, we hypothesize that a shelter’s LOS may exhibit significant variation over calendar periods; that such variation may be accurately computed and disentangled from the mix of incoming animal sizes and ages; and that calendar-period LOS can thus be informative to shelters.

We propose LOS computations that are appropriate for assessing and comparing shelter operations across calendar periods. The study shows how KM fits [35] can be used for the LOS distribution over fixed calendar periods rather than specific animal cohorts; how these enable comparisons; and how Cox proportional hazards regressions [36] can similarly test for changes in the LOS distribution from one period to another while accounting for animal characteristics. KM and Cox refer to survival analysis, but they apply broadly to time-to-event studies, including in veterinary epidemiology [22], and have already been used for LOS of shelter animals [21]. In this article we avoid the term *survival* as it would be confusing in the animal sheltering context.

The current shelter practice (which we label **HistLOS**) and our proposed corrected formulation (which we label **ExitLOS**) use standard statistical methods presented in the next section. These are then illustrated with the exploratory analysis of dog ExitLOS in a large shelter in Orange County, California, for periods with duration ranging from a month to well over a year. The analysis shows that there are statistically significant longitudinal changes in ExitLOS, suggesting that it is a useful indicator in shelter operational decisions. The article aims to demonstrate the conceptual soundness of ExitLOS and illustrate its operational value.

## 2 Materials and methods

### 2.1 LOS definitions and Kaplan-Meier computation

We use standard shelter terminology of **intakes** denoting animals coming into the shelter; and **outcomes** animals leaving the shelter. We define LOS to include the intake and outcome date, to account for shelter care needed even for same-day outcomes [27]. Hence a same-day arrival and departure correspond to LOS = 1.

We use Kaplan-Meier (KM) time-to-event [35] fits to compute the LOS distribution non-parametrically, with any outcome (end of shelter stay) considered an event. The result is shown as a KM curve – the fraction of the animals remaining in the shelter as a function of elapsed LOS (number of days). The term *survival function* is commonly used for this curve in statistical terminology, but here we use the terms **KM stay function** or just **KM curve** to avoid confusion in the animal sheltering context. The KM curve starts at 1.0 for time t = 0 (all animals entering the shelter) and its value at t = 1 is the fraction of incoming animals that are still in the shelter at the end of the intake date.

#### 2.1.1 HistLOS.

We use the label **HistLOS** for the LOS computation historically used by shelters, counting only LOS of outcomes that occurred in a period of interest. Without further adjustments, the KM fit in this case captures the distribution of observed LOS values. As we argue in the introduction (and reinforce with the first set of results), HistLOS is misleading, and we address its shortcomings by using truncation and censoring to restrict KM to sharply defined calendar periods.

#### 2.1.2 ExitLOS via truncation and censoring.

**Fig 1:**
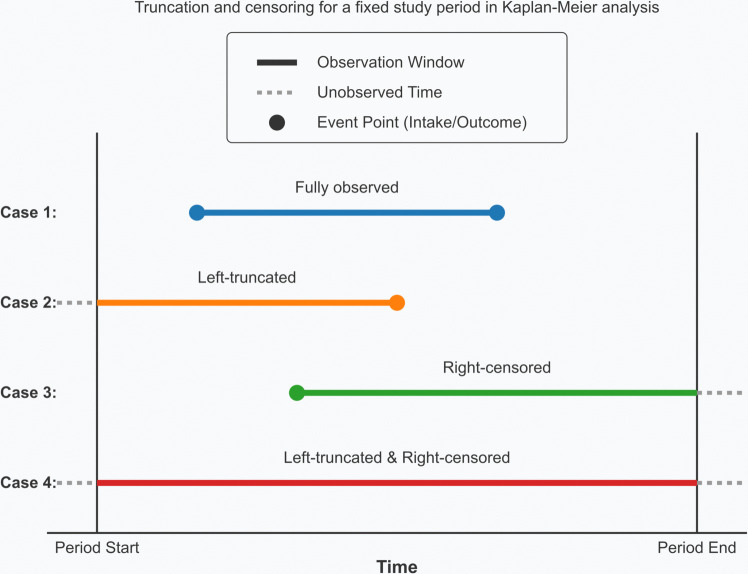
Left-truncation and right-censoring in KM and Cox fits. For a fixed study period (start to end), an individual’s observation window (intake to outcome) falls into one of four cases. Only the portion of the stay that overlaps with the study period is used in the KM regression. However, the elapsed LOS is properly counted from intake.

KM provides a mechanism for properly accounting for all resident animals, and their departures or lack thereof. For a fixed study period (start to end), an individual’s observation window (intake to outcome) falls into one of four cases (Fig 1):

Fully observed — arrives after study period starts, departs before period ends

Left-truncated — is already resident when study period starts, departs before period ends

Right-censored — arrives after study period starts, is still resident when period ends

Both left-truncated and right-censored — is resident throughout the study period

For left-truncated individuals, only the portion of the stay that overlaps with the study period affects the fit. However, the elapsed LOS is computed from intake, i.e., does not reset at the start of the study period. For right-censored individuals, the eventual LOS is considered unknown but exceeds the elapsed LOS as of the end point of the study period. The fact that the individual did not depart from the shelter during (and all the way to the end of) the study still affects the KM fit. We use the label **ExitLOS** for this computation, because it is based on the probability of exit from the shelter at each time point.

KM assumes that censorship is non-informative, i.e., censored observations have the same outcome prospects as observations followed to their outcomes. The censoring applied in ExitLOS for dogs remaining in the shelter at the end of a study period is determined by arbitrary calendar transitions (end of month or quarter) and is not directly connected to any event specific to that dog. However, the timing of operational changes like adoption fairs or staff vacation may be associated with calendar boundaries. We assume that such association is weak, so that non-informative censoring is a reasonable approximation. We make the same assumption for truncation at the beginning of a period.

### 2.2 Other statistical methods

In plots, we test the difference between the KM curves at each time point using Klein’s method [37]. The Klein test is not used as the primary statistical comparison, but rather as an extension of the CI bands: An accessible way to clarify the CI overlap of the two KM curves and identify the contiguous region in which there is clear separation between KM curves.

To compare two KM curves, we use the Cox proportional hazards method [36] with the Breslow baseline estimator [38] and Efron [39] tie-breaking. (Ties are common as we measure shelter LOS in integer days.) Cox accommodates left-truncation and right-censoring. For pairwise statistical testing, we use the Cox score, which obviates the need for a log-rank test. (The survival package in R and the lifelines module in Python do not support log-rank tests with left-truncation; for comparing two groups, the Cox score is a readily available alternative with equivalent P values.) We recognize that within-animal correlation of observations across period boundaries may exist and could be addressed via clustered standard errors. However, we make the simplifying approximation of treating the observations in different periods as independent.

We compute the standard errors of KM means via Greenwood’s formula [40]. As our KM curves are heavy tailed, we also report 90^th^ stay percentiles, i.e., the time point at which only 10% of the animals remain in the shelter.

To remove the effect of changes in the size and age distribution of incoming dogs on the Cox hazard ratio, we stratify the Cox regression by (categorical) size and age. With stratification, each size and age combination gets its own baseline hazard, but the period has the same fixed proportional effect.

When we examine the hazard ratio of two periods, we visualize the hazard proportionality assumption via Schoenfeld residuals [41]. We test the slope of the residuals, with slope = 0 as the null hypothesis [42]. In this test, a non-significant result is consistent with proportionality. We further visually check deviance residuals [43] for outliers. Finally, we inspect log cumulative hazard plots against log time, for which proportional hazards should produce roughly parallel lines.

Tests are done at the 0.05 significance level, and 95% confidence intervals (CI) are shown – including in graphical form.

### 2.3 Software

KM and Cox are available in most statistical software packages. We use the *lifelines* Python module (version 0.30.0) [44], with Python 3.11.12 (some module versions: pandas 2.2.3; matplotlib 3.10.3) and Spyder IDE 6.0.7 on MacOS 15.5. We also use the *survival* package version 3.7.0 [45] in the R language version 4.4.2 [46] on RStudio 2025.09.1 + 401 for some additional capabilities, namely Cox diagnostics with left-truncated observations.

### 2.4 Data

Animal-level intake and outcome data for dogs were provided by Orange County Animal Care [47] (abbreviated as OC), serving the majority of Orange County, California.

We discarded outcome types ‘DELETE DUP’, ‘FOUND EXP’, ‘HOME EXP’, ‘LOST EXP’, ‘SURG SCHED’, ‘SURG WAIT’ and intake types ‘DISPO REQ’, ‘EUTH REQ’, and ‘WILD’, as these do not represent regular live admissions to the shelter. (This filter drops internally inconsistent entries, as it is not practical to investigate them individually.) For HistLOS and ExitLOS, we use all remaining data and do not differentiate by outcome type.

We study only dogs, in calendar periods of one or more whole months from July 2018 to June 2024 (the shelter’s Fiscal Years 2018–2019–2023–2024). Length of stay is capped to 550 days, this cap being operative in 11 out of >27,000 dogs. (The very longest residents are de facto outliers, likely to garner individualized attention by staff and volunteers.) Mean LOS values presented in this study denote restricted means up to this cap.

We make limited use of animal age and size. The age, computed from date of birth to intake date, is rounded up to integer years and constrained to an upper bound of 15, to avoid categories with very few datapoints. The size categories are X-LRG, LARGE, MED, SMALL, TOY, and PUPPY. (The use of PUPPY as a size category may be counter-intuitive, but it is common, perhaps reflecting uncertainty about a puppy’s eventual size.) A total of 51 dogs with size PUPPY but age greater than 1 year were reclassified as MED. Size and age are determined at intake and thus do not vary over a dog’s stay in the shelter.

## 3 Results

We present three sets of results. The first, a comparison of just two consecutive months, illustrates how misleading the LOS currently practiced in shelters can be. The second, a sequence of 24 quarterly periods, shows that statistically significant changes in LOS occur often, and their magnitude is consequential for shelter resources. Finally, we factor some basic dog characteristics in a comparison of two prolonged periods before and after the COVID-19 pandemic (which entailed changes in shelter operations), to show that LOS changes may be an indicator of the impact of shelter operational decisions. The variety in the results is intended to illustrate a subset of the possible uses of LOS by shelters.

### 3.1 October-November 2023

As an initial demonstration, we compare KM curves for October and November 2023. In the Fall of 2023, OC was accumulating long residents. In early November there was a fire near the shelter. As the shelter was obliged to implement temporary but drastic operational changes, national organizations assisted by transferring over 100 dogs (1/3 of the resident dogs at the time) out of OC. This substantial increase in outcomes from October to November accentuates the differences in LOS computation.

#### 3.1.1 HistLOS KM.

**Fig 2:**
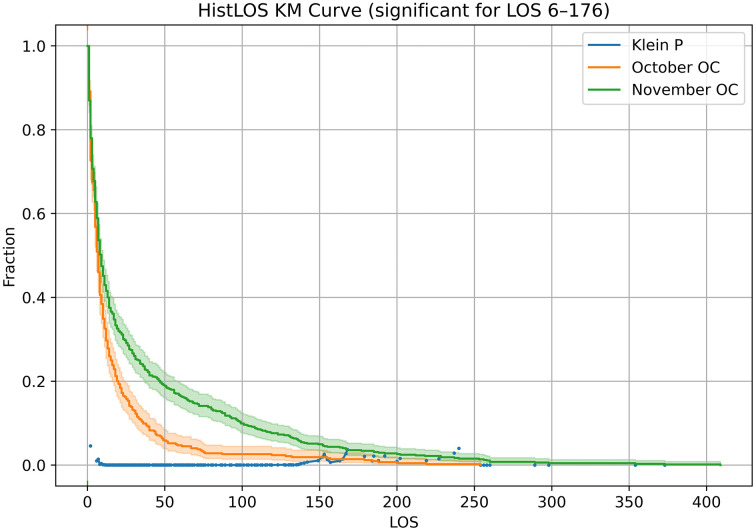
HistLOS KM curves for October and November 2023. KM curves with 95% CI bands show longer stays for November than October, an artifact of many long residents exiting in November. Klein tests (shown when P < 0.05) aid in identifying the region of separation between curves; an overall Cox score test is presented later. HistLOS includes the full stay of any dogs with in-period outcomes, with no truncation or censoring.

HistLOS, reflecting the metric used by shelters, just computes intake-to-outcome LOS for all dogs with outcomes in each period. Its basic statistics are in Table 1 for the two-month period and each separate month. HistLOS KM curves in Fig 2 indicate that November has longer stays. From October to November, the mean HistLOS rises from 16.1 to 32.3. (Here and in other KM fits, we do not show medians, as they fall within the rapid initial drop in the curves, which is less informative.) The Klein test [37] serves as an auxiliary to the CI bands, showing the contiguous region of clear separation between the two KM curves. Here, the test yields P < 0.05 for stays from 6 to 176 days (and intermittently outside this interval).

**Table 1:** HistLOS statistics for October and November 2023.

period	count	mean	standard deviation	min	max
**Unified** ^*^	1,069	25.9	48.0	1	409
**October**	424	16.1	30.3	1	254
**November**	645	32.3	55.7	1	409

Statistics are for the stay (in days) from intake to outcome. HistLOS includes the full stay of any dogs with in-period outcomes, with no truncation or censoring. Longer HistLOS in November is an artifact of exiting long residents.

* The Unified section treats the data as a single two-month period.

#### 3.1.2 ExitLOS.

Descriptive statistics of ExitLOS stay, i.e., days from intake to outcome or censoring, are in Table 2 for the two-month period and each separate month.

**Table 2:** Descriptive ExitLOS stay statistics for October and November 2023.

Truncation/ Censoring	count	mean	standard deviation	min	max
Unified^*^ **October–November period**					
**All**	1,229	27.8	51.4	1	409
**Not Truncated**	933	9.0	10.0	1	61
**Left-Truncated**	296	87.4	77.4	2	409
**Not Censored**	1,069	25.9	48.0	1	409
**Right-Censored**	160	41.0	68.8	1	398
**Not Truncated & Not Censored**	805	8.5	9.4	1	60
**Not Truncated & Right-Censored**	128	12.0	13.0	1	61
**Left-Truncated & Not Censored**	264	78.9	73.0	2	409
**Left-Truncated & Right-Censored**	32	157.1	78.6	63	398
**October period**					
**All**	723	35.7	56.9	1	395
**Not Truncated**	427	7.6	7.0	1	31
**Left-Truncated**	296	76.1	71.2	2	395
**Not Censored**	424	16.1	30.3	1	254
**Right-Censored**	299	63.4	72.2	1	395
**Not Truncated & Not Censored**	291	5.9	5.2	1	26
**Not Truncated & Right-Censored**	136	11.3	8.8	1	31
**Left-Truncated & Not Censored**	133	38.3	46.4	2	254
**Left-Truncated & Right-Censored**	163	106.9	73.1	32	395
**November period**					
**All**	805	34.0	58.6	1	409
**Not Truncated**	506	6.5	5.8	1	30
**Left-Truncated**	299	80.7	75.8	2	409
**Not Censored**	645	32.3	55.7	1	409
**Right-Censored**	160	41.0	68.8	1	398
**Not Truncated & Not Censored**	387	5.7	5.0	1	27
**Not Truncated & Right-Censored**	119	9.0	7.3	1	30
**Left-Truncated & Not Censored**	258	72.2	71.3	2	409
**Left-Truncated & Right-Censored**	41	133.8	82.4	37	398

Statistics are for the stay (in days) from intake to outcome or censoring. For each period, ExitLOS includes all dogs in the shelter’s care for any fraction of the period, with truncation and censoring as illustrated in Fig 1.

* The Unified section treats the data as a single two-month period.

In ExitLOS, (left-)truncation means the dog was already resident at the beginning of the period, while (right-)censoring means the dog was still resident at the end of the period. These are in effect the counts of resident dogs at period boundaries. Accordingly, the number of censored observations for October equals the number of truncated observations for November. Outcomes (dogs exiting the shelter) in each period equal ExitLOS non-censored observations (or total observations of HistLOS in Table 1). A period’s intakes equal ExitLOS non-truncated observations. For ExitLOS, the Unified period has fewer observations than the sum of the two months. The difference is 299, exactly the censored observations in October or truncated observations in November. The 299 dogs in shelter care at the period boundary generate observations in both months.

**Fig 3:**
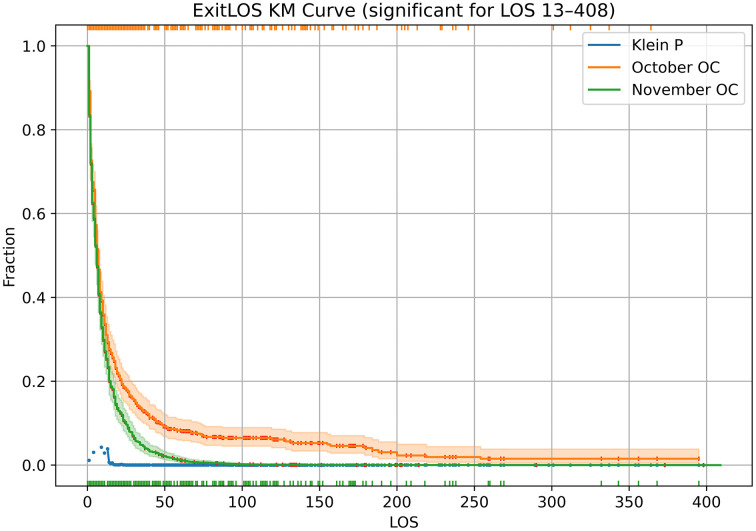
ExitLOS KM curves for October and November 2023. KM curves with censoring marks and 95% CI bands show LOS shrunk from October to November, because of a spike in transfer outcomes in November. Truncation marks are (color coded) at the top and bottom. Klein tests (shown when P < 0.05) aid in identifying the region of separation between curves. (An overall Cox score test will be presented in Table 3) ExitLOS uses data on dogs in the shelter for any part of the study period but (left-)truncates and (right-)censors at the boundaries of the period (Fig 1).

The KM curves for ExitLOS (Fig 3) show the opposite trend from HistLOS (Fig 2). This reflects the fact that there were fewer outcomes in October, with accumulation of long residents; and more outcomes in November, with departure of long residents (Table 2). The 95% CIs in Fig 2 suggest the change is significant. The Klein test consistently yields P < 0.05 for stays of 13 days or higher, i.e., after the initial rapid drop of both curves.

**Table 3:** Statistical comparisons of the KM curves of two monthly periods, for each type of LOS computation.

	October		November		Cox score^*^	
	mean	standard error	mean	standard error	z statistic	P value^+^
**HistLOS**	16.1	1.5	32.3	2.2	−5.5	< 0.0001
**ExitLOS**	27.7	4.1	10.1	0.6	7.6	< 0.0001

Means (and their standard errors based on Greenwood’s formula [40]) are derived from the KM curves shown in Figs 2–3.

* The Cox score compares the distributions, not the means.

+ The actual P values are 2.9x10^–8^ for HistLOS and 3.5x10^–14^ for ExitLOS, and they equal those of a log-rank χ² test for a two-level comparison.

#### 3.1.3 Additional statistical tests.

The KM plot CI and Klein tests show HistLOS and ExitLOS changes in opposite directions. This is further confirmed by additional statistics in Table 3. From October to November, mean HistLOS rises from 16.1 to 32.3, but mean ExitLOS drops from 27.7 to 10.1. These means (and their standard errors) confirm the visual impression of the KM curves (Figs 2–3). The statistical comparison of the two periods uses the Cox score. The changes in ExitLOS and HistLOS are highly significant (P < 0.0001) but in opposite directions. (It’s apparent from the means and their standard errors that the difference of the means is also significant, but that would be a narrower test, not the same as testing the KM curve itself.)

#### 3.1.4 Descriptive statistics in relation to KM.

HistLOS rose from October to November. Considering the statistics in Table 2, this rise is misleading. The higher values in November reflect the transfer of long residents out of the shelter. The lower value in October occurs because long residents are not included in HistLOS while they continue to reside in the shelter. Table 2 shows that out of 296 dogs in the shelter when October began, the majority (163) are still there at the end; out of 427 that came in during the month, only 136 are still resident at the end. For November, out of 299 initial residents only 41 are still there at the end; and out of 506 that came in, 119 are still resident at the end. This shows that outcomes in November were faster than October overall, and much faster for longer residents. HistLOS disregards animals that remain in the shelter through the end of the study period – and is higher when outcomes accelerate during a period.

This example shows that HistLOS – the only LOS used in shelters and supported by shelter databases – is problematic. When there are longitudinal changes in intake and outcome patterns (whether caused by external or internal factors), HistLOS produces a misleading comparison of periods, because it fails to properly attribute the LOS of animals whose stay crosses period boundaries. ExitLOS provides a rigorous computation of each period’s LOS and allows the detection and assessment of changes.

Note that a KM fit for the unified two-month period (top section of Table 2) is identical to a KM fit obtained by aggregating (stacking) the observations of the two monthly periods (middle and bottom section of Table 2) and produces identical confidence intervals, even though a dog that crosses the period boundary is represented by two observations in the stacked fit and only one in the unified fit.

### 3.2 KM statistics for a sequence of quarterly periods

We analyze ExitLOS from July 1, 2018, to June 30, 2024 (six years) in 24 quarterly periods. Each period is labelled by its starting month (e.g., 20−07 for July-September 2020). Descriptive statistics are shown in Table 4. In the Unified section of Table 4, truncation and censoring are rare, because most observations involve stays entirely within the six-year unified period. The 147 left-truncated observations are the dogs in the shelter at the very beginning, and the 258 right-censored the ones remaining in the shelter at the very end. The overall mean stay is 17.1 days, but truncated or censored observations involve much longer stays.

**Table 4:** Descriptive statistics of quarterly ExitLOS observations, July 1, 2018 – June 30, 2024.

	Unified^*^ (as single period)			Aggregated^+^ (summed over periods)			Average^§^ (per period)
	count	mean	std	count	mean	std	count
**All**	28,059	17.1	38.9	32,758	22.9	49.9	1,365
**Not Truncated**	27,912	16.7	37.4	27,912	10.7	14.0	1,163
**Left-Truncated**	147	98.1	130.9	4,846	93.2	99.4	202
**Not Censored**	27,801	16.9	38.6	27,801	16.9	38.6	1,158
**Right-Censored**	258	47.5	58.6	4,957	56.7	82.2	207
**Not Truncated & Not Censored**	27,654	16.4	37.0	23,917	8.5	10.1	997
**Not Truncated & Right-Censored**	258	47.5	58.6	3,995	23.9	23.6	166
**Left-Truncated & Not Censored**	147	98.1	130.9	3,884	68.5	83.1	162
**Left-Truncated & Right-Censored**				962	192.7	97.7	40

Statistics are for the stay (in days) from intake to outcome or censoring, for the period July 1, 2018, to June 30, 2024, with ExitLOS truncation and censoring as illustrated in Fig 1. Between the Unified and Aggregated sections, the number of non-truncated observations is identical (27,912) and equals total intakes. Likewise, the number of non-censored observations (27,801) matches and equals total outcomes.

* The Unified section has one observation per dog for the whole six-year unified period.

+ In the Aggregated section, the data is partitioned into 24 quarterly periods, each including all dogs in the shelter’s care for any fraction of the period. Truncation and censoring occur period-by-period, but the statistics are then aggregated over all periods.

§ The Average column shows per-period counts (the counts from the Aggregated section divided by 24), which are easier to relate to shelter counts of intakes, outcomes, and residents.

In the Aggregated section of Table 4, the additional 4,699 observations (32,758–28,059) are the dogs whose stay crossed period boundaries. In the Average count section, truncated ExitLOS observations equal the beginning resident count per quarter, averaging 202. Censored ExitLOS equal final resident count, averaging 207. Non-censored ExitLOS observations equal total outcomes, averaging 1,158 per quarter. Finally, truncated & censored ExitLOS equal dogs in the shelter for an entire calendar quarter: on the average, of 202 dogs in the shelter at the beginning of a quarter, 40 were still there at the end of the quarter.

**Fig 4:**
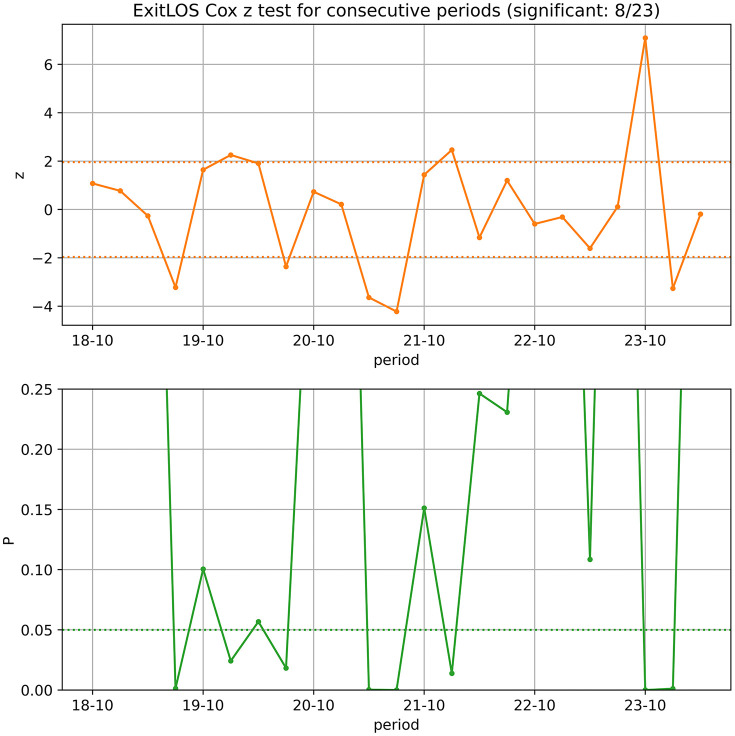
Cox scores of ExitLOS for consecutive quarterly periods. These are computed from quarterly ExitLOS periods. A test is labelled by the starting month of the second period in the pair, e.g., 20−10 for the comparison of October-December 2020 to July-September 2020. The Cox z statistic is in the top subplot and the P value in the bottom. Positive z corresponds to faster outcomes for the chronologically later period. Dashed lines mark the 0.05 significance level. Eight of 23 changes are statistically significant (P < 0.05), which suggests that the quarter-to-quarter variation is not just noise.

The main purpose of this example is to show that LOS changes on a quarterly time scale are detectable – at least for a shelter the size of OC or larger. We compute Cox scores to test for differences in consecutive quarters (Fig 4). We do not report hazard ratios, as Cox is not used to model LOS quantitatively but only to test whether the KM curves differ.

Of 23 quarterly comparisons, 8 (35%) have P < 0.05. In lieu of False Discovery Rate correction, we simply do an exact one-sided binomial test. With success probability 5% in the null hypothesis, a binomial test of 23 trials and 8 successes (35%) rejects the null hypothesis with P < 0.0001. Of the 8 significant quarterly changes, 20−07 is associated with COVID-19 lockdown effects, 23−10 with the fire-related transfer spike of November 2023, and 24−01 with a rebound from these transfers. If we exclude these three as obvious exogenous changes, a binomial test with 20 trials and 5 successes (25%), rejects the null hypothesis with P = 0.0026. We also test each quarter against the same quarter a year before. Of 20 such comparisons, 10 (50%) have P < 0.05. This suggests that, though some seasonality may exist, the consecutive-quarter effects are not a side-effect of seasonal cycles.

**Fig 5:**
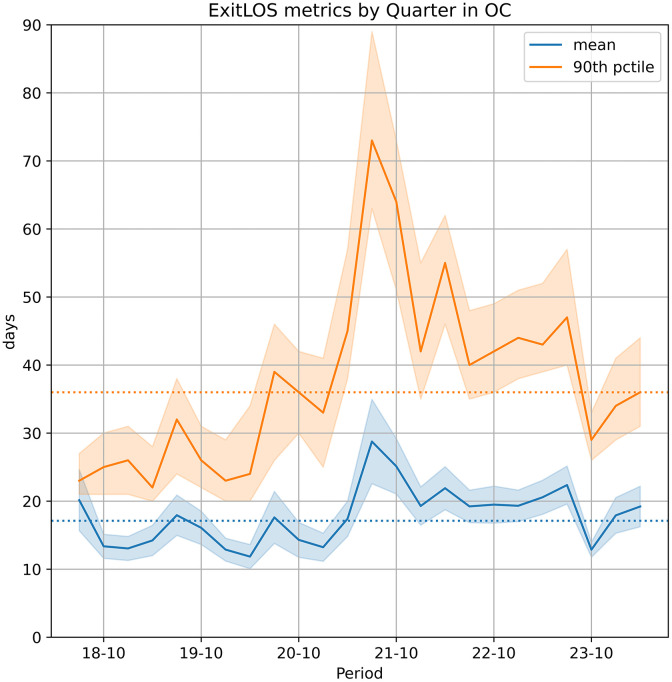
Means and 90^th^ stay percentiles of ExitLOS by quarterly period. These are computed, with 95% CI bands, from quarterly ExitLOS KM fits from July 1, 2018, to June 30, 2024, with periods labelled by their starting month (e.g., 20−07 for July- September 2020). For the 90^th^ stay percentile, the CI band is computed by inverting the 95% CI bands of the KM curves. Dashed lines show the mean (17.1) and 90^th^ stay percentile (36) for a KM aggregate curve covering the entire six-year period. (These are not the arithmetic means of the quarterly values.) The changes in LOS are operationally consequential as mean LOS has a proportional impact on housing and staffing needs.

The results demonstrate that a period-by-period ExitLOS analysis is likely detecting real changes, whether exogenous or endogenous. For more tangible metrics, we compute the quarterly means and 90^th^ stay percentiles (the time by which 90% of animals have left and 10% are still in care) of the KM curves. These are shown in Fig 5. Until the middle of 2021, mean LOS fluctuates around 15 days, though the 90^th^ percentile shows a slow rising trend. This is followed by a sharp increase in LOS and a partial recovery, but with LOS remaining above pre-2021 levels until the large number of transfers in quarter 23−10 (the same event that drives the change in Fig 3).

The longest ExitLOS occurs in 21−07, with mean 28.8 (95% CI 22.6–34.9) and 90^th^ stay percentile 73 (95% CI 63–89). The shortest ExitLOS occurs in 20−04, with mean 11.8 (95% CI 10.1–13.6) and 90^th^ stay percentile 24 (95% CI 20–34).

In these quarterly periods, the median LOS ranges from 5 to 8 days; the ratio of 90^th^ percentile to median ranges from 3.7 to 9.2. (For reference, this ratio is approximately 3.3 for a geometric distribution.) While most dogs have quick outcomes, the tail of the distribution is where operational stress and welfare risks accumulate. For 19−07, the mean is 17.9 (95% CI 15.0–20.9) and the 90^th^ stay percentile is 32 (95% CI 24–38). In 22−01 (after the peak of 21−07), the mean settles back to 19.3 (95% CI 16.5–22.1), but the 90^th^ stay percentile stays distinctly higher at 42 (95% CI 35–55). This shows an accumulation of long-stay dogs. The sharp drops in 23−10, with a reduction in the CI span, are a consequence of the exogenous large number of transfers, induced by a nearby fire, as discussed in the comparison of October and November 2023 (Fig 3). The mean ExitLOS for the entire period is 17.1 days and the 90^th^ percentile is 36 (computed from KM for the entire six-year period, not arithmetic means of the quarterly values). Other things being equal, a change in ExitLOS translates proportionally to a change in shelter resident count, which in turn impacts all aspects of care [1]. From 22−01 to 22−04, mean ExitLOS rises by 13.6% (from 19.3 to 21.9) which implies proportional increases in dog housing use and needed staffing (animal care attendants), though it is not statistically significant. The appropriate operational response to an ExitLOS change may depend not only on whether the change is significant but also on how constrained the shelter’s capacity is to start with.

Thus, ExitLOS shows differences across periods, but not a steady trend. Frequent re-computation is important for bringing LOS up to date. A statistically significant shift may or may not be operationally important. It may be too small to matter; or attributable to unusual events (such as hoarding cases or the spike in transfers in November 2023). But detecting and measuring the change is the necessary first step. The variation of LOS from one calendar period to another may also be the result of changes in incoming dog characteristics such as size and age. We examine these variables in a comparison of two longer periods in the next section, alongside operational restrictions which may explain the observed LOS increase.

### 3.3 Extended periods before and after COVID-19 restrictions

We take a closer look at two extended periods on either side of the COVID-19 pandemic. This comparison is motivated by the fact that OC used different visitation and adoption systems post-COVID-19, which affected adoption rates [48,49] and may have therefore impacted LOS. Because OC moved from antiquated to modern facilities in the spring of 2018, we define the pre-COVID-19 period as July 1, 2018 – February 29, 2020 (20 months). We do not study the main COVID-19 period that entailed rapid changes induced by national and local policies. Since other shelters gradually relaxed COVID-related restrictions in 2021 (e.g., Los Angeles Animal Services re-admitted the public to kennel areas in September 2021) we conservatively exclude all of 2021. We set the post-COVID-19 period to January 1, 2022 – October 31, 2023 (22 months) to exclude the fire near the OC shelter which (as previously mentioned) induced temporary operational changes in November 2023. The inequality of the lengths of the periods does not affect the LOS analysis.

While regionally shelters gradually re-admitted the public to kennel areas in 2021, OC carried substantial access restrictions into the post-COVID-19 period [48,49] and did not fully return to the pre-COVID-19 visiting system until 2025 [50]. Thus, the comparison of these two periods entails two different visitor access regimes, but without the externalities of the March 2020 – December 2021 COVID-19 tumult or the late 2023 local fire disruption. Descriptive statistics are in Table 5. We omit the min and max, which are not informative for long study periods, but include a breakdown by age and size.

**Table 5:** Descriptive statistics of ExitLOS observations for pre- COVID-19 and post-COVID-19 periods.

	pre-COVID-19^*^				post-COVID-19^+^			
	count	percent	mean	std	count	percent	mean	std
**All**	9,641		14.8	36.5	8,719		21.3	44.5
**Not Truncated**	9,494	98.5%	13.5	31.3	8,503	97.5%	18.5	36.5
**Left-Truncated**	147	1.5%	98.1	130.9	216	2.5%	132.7	122.1
**Not Censored**	9,442	97.9%	13.5	32.3	8,420	96.6%	19.8	42.5
**Right-Censored**	199	2.1%	75.3	106.0	299	3.4%	63.4	72.2
**Not Truncated & Not Censored**	9,295	96.4%	12.2	26.0	8,204	94.1%	16.9	33.4
**Not Truncated & Right-Censored**	199	2.1%	75.3	106.0	299	3.4%	63.4	72.2
**Left-Truncated & Not Censored**	147	1.5%	98.1	130.9	216	2.5%	132.7	122.1
**Left-Truncated & Right-Censored**	0	0.0%			0	0.0%		
**Size PUPPY**	889	9.2%	5.4	5.7	1,234	14.2%	7.8	14.0
**Size TOY**	416	4.3%	14.7	17.2	129	1.5%	6.2	7.7
**Size SMALL**	2,479	25.7%	9.8	14.2	2,194	25.2%	9.5	11.2
**Size MED**	2,439	25.3%	8.8	13.7	1,813	20.8%	10.4	14.6
**Size LARGE**	3,304	34.3%	25.3	57.2	3,217	36.9%	40.7	65.6
**Size X-LRG**	114	1.2%	22.0	52.7	132	1.5%	36.8	63.8
**Age 1**	1,621	16.8%	6.7	8.1	2,037	23.4%	10.8	21.2
**Age 2**	1,290	13.4%	12.2	23.8	1,336	15.3%	23.1	42.9
**Age 3**	1,530	15.9%	16.1	35.7	1,482	17.0%	28.8	52.3
**Age 4**	1,046	10.8%	18.2	46.5	819	9.4%	27.1	50.9
**Age 5**	853	8.8%	20.8	50.6	630	7.2%	30.6	61.3
**Age 6**	744	7.7%	23.3	55.6	559	6.4%	29.2	55.2
**Age 7**	459	4.8%	22.1	52.0	296	3.4%	28.8	62.7
**Age 8**	409	4.2%	15.3	26.3	248	2.8%	26.8	54.0
**Age 9**	473	4.9%	15.0	39.6	355	4.1%	18.6	38.2
**Age 10**	248	2.6%	13.4	30.7	140	1.6%	21.2	59.1
**Age 11**	380	3.9%	13.4	31.5	303	3.5%	12.8	23.0
**Age 12**	131	1.4%	11.3	22.6	83	1.0%	10.4	13.0
**Age 13**	186	1.9%	11.0	17.9	159	1.8%	9.2	14.2
**Age 14**	107	1.1%	11.1	21.8	112	1.3%	7.6	10.3
**Age 15**	164	1.7%	5.3	8.2	160	1.8%	5.0	7.3

Any dog resident at any point in a period is an observation, with truncation and censoring as illustrated in Fig 1. Mean and standard deviation are for the stay from intake to outcome or censoring. Age in integer years (rounded up and capped at 15) is computed from date of birth to intake date.

* The pre-COVID-19 period is July 1, 2018 – February 29, 2020.

+ The post-COVID-19 period is January 1, 2022 – October 31, 2023.

Recalling that non-truncated observations equal intakes, we see that intakes drop from 9,494 total (474 per month) pre-COVID-19 to 8,503 total (387 per month) post-COVID-19. The total number of observations is accordingly smaller post-COVID-19. Non-censored observations equal outcomes (dogs leaving the shelter) and show a similar reduction from 9,442 (472 per month) to 8,420 (383 per month). However, for nonlive outcomes (not shown in the table) there’s an increase from 336 pre-COVID-19 to 507 post-COVID-19. In the post-COVID-19 period, there are more left-truncated and more right-censored observations (by count or percentage), indicating more dogs present in the shelter at the beginning and end of the period (consistent with elevated LOS).

The post-COVID-19 period shows an increase in puppy size percentage from 9.2% to 14.2% and a decrease in medium size from 25.3% to 20.8%. Related to this, by age, there’s an increase in age 1 from 16.8% to 23.4%, smaller increase in ages 2 and 3, small decreases in ages 4–13, and small increases in ages >13. There may be additional changes in the joint distribution of size and age, which we do not examine here.

**Fig 6:**
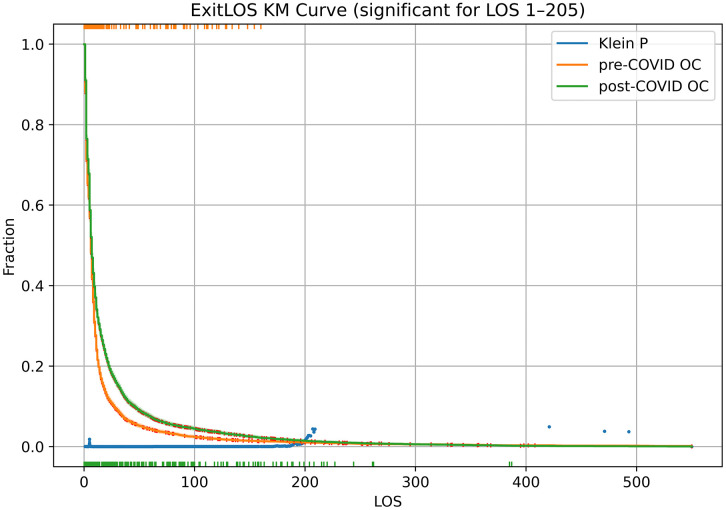
KM curves for the pre-COVID-19 and post-COVID-19 periods. The pre-COVID-19 period is July 1, 2018 – February 29, 2020. The post-COVID-19 period is January 1, 2022 – October 31, 2023. KM curves are for ExitLOS, with (left-)truncation and (right-)censoring at the boundaries of the period (Fig 1). Censoring marks on the KM curves and 95% CI bands are shown. Truncation marks are (color coded) at the top and bottom. The P values of Klein time-point tests are shown when P < 0.05. There is clear separation between the two periods for all time points up to 205.

The KM curves for the two periods are shown in Fig 6. The 95% CI visually suggest the difference is significant; the Klein test yields P < 0.05 for all time points from 1 to 205, by which point 99.1% of dogs pre-COVID-19 and 98.8% post-COVID-19 leave the shelter. The statistics of the KM fits are in Table 6. From a pre-COVID-19 mean of 15.1 (95% CI 14.3–16.0), ExitLOS rose to 20.8 (95% CI 19.8–21.7) post-COVID-19. The 90^th^ percentiles of the KM curves show a larger increase, from 27 (CI 26–29) to 46 (CI 42–48). All metrics suggest that dogs are staying in the shelter longer in the post-COVID-19 period.

**Table 6:** KM results for the pre-COVID-19 and post-COVID-19 periods.

	pre-COVID-19^*^	post-COVID-19^+^
**mean**	15.1	20.8
**standard error of mean**	0.43	0.49
**CI of mean**	14.3–16.0	19.8–21.7
**90th percentile**	27	46
**CI of 90th percentile**	26–29	42–48

All statistics are in days. KM fits are for ExitLOS, with (left-)truncation and (right-)censoring at the boundaries of the period.

* The pre-COVID-19 period is July 1, 2018 – February 29, 2020.

+ The post-COVID-19 period is January 1, 2022 – October 31, 2023.

To refine the comparison, we do two Cox proportional hazards regressions, one without stratification (therefore directly corresponding to the KM fits) and another with size and age strata. The second Cox regression in effect assumes a different non-parametric baseline for each combination of size and age, but with the effect of the period being fixed on all baselines. The results are in the top section of Table 7, and show hazard ratios of 0.812 and 0.804, statistically significant with z –13.8 and –13.9 (P < 0.0001). The Cox score without stratification essentially tests for difference in the KM curves of Fig 6.

**Table 7:** Cox proportional hazards regressions comparing the post-COVID-19 to the pre-COVID-19 period.

	no strata	age & size strata
**hazard ratio** ^*^	0.812	0.804
**hazard ratio CI**	0.788–0.836	0.779–0.829
**log hazard ratio**	−0.0904	−0.0950
**standard error** ^**+**^	0.0151	0.0157
**Cox Score Test**		
**z statistic**	−13.8	−13.9
**P value**	< 0.0001	< 0.0001
**Non-Proportionality Test** ^**¶**^		
χ^2^ **statistic**	0.34	0.05
**degrees of freedom**	1	1
**P Value**	0.56	0.82

These are comparisons of the post-COVID-19 period (January 1, 2022 – October 31, 2023) with pre-COVID-19 as the referent (July 1, 2018 – February 29, 2020).

* Cox hazard ratio statistics (with 95% CI).

+ The standard error refers to the log hazard ratio.

§ The non-proportionality test is applied to Schoenfeld residuals, with zero slope (proportionality of hazards) as the null hypothesis.

**Fig 7:**
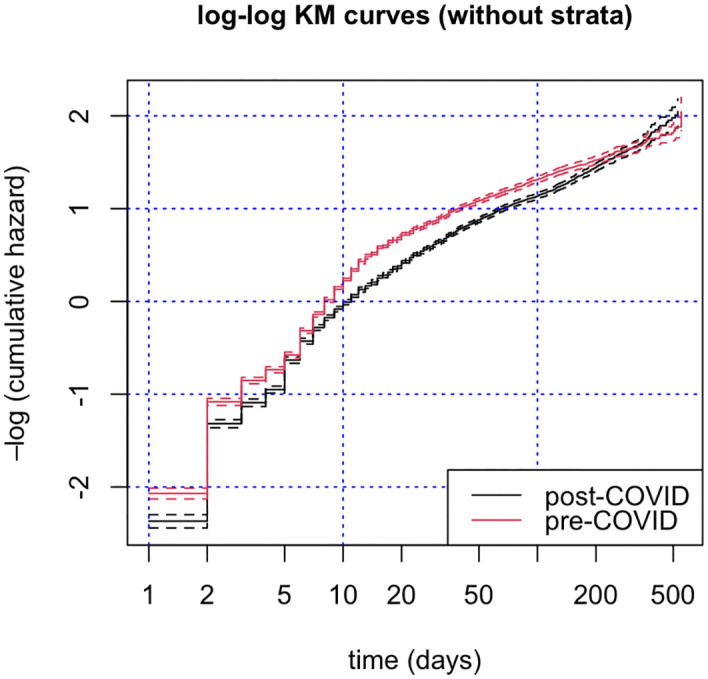
Log cumulative hazard plot for the pre-COVID-19 and post-COVID-19 periods. The pre-COVID-19 period is July 1, 2018 – February 29, 2020; the post-COVID-19 period is January 1, 2022 – October 31, 2023.

The negative log of the KM curve is the cumulative hazard. If the proportional hazards assumption holds, the log cumulative hazards of the two periods should move in parallel when plotted against log time. This plot for the Cox regression without stratification is shown in Fig 7. There is a tightening around 6 days and the lines eventually cross around 300 days. At the right end of the time axis, the shelter may be taking additional steps to obtain outcomes because, even in a large shelter, the very longest residents are likely to gain attention. Thus, proportional hazards hold reasonably well for short-to-medium stays but may not capture dynamics for animals staying >200 days. The Klein test (Fig 6) similarly shows that the gap in stay fractions is statistically significant up to 205 days.

This log cumulative hazard plot is not useful for the stratified regression, as each age and size combination yields a separate pair of lines. Instead, we visually check the Schoenfeld residuals [41] (provided as supporting information S1 Fig). Some oscillation exists, accentuated at very long times (>200 days, as noted for the cumulative hazard in Fig 7). The behavior of the residuals suggests imperfect but workable proportionality for all but the longest stays. In the bottom section of Table 7, we test the slope of the Schoenfeld residuals (with slope = 0 as the null hypothesis [42]). The P values of 0.56 (without strata) and 0.82 (with size and age strata) do not reject the null hypothesis and are thus consistent with proportionality of hazards for both models.

We finally plot the deviance residuals [43] of the stratified model (provided as supporting information S2 Fig). These have the desired distribution, visually symmetric about zero. Two clouds of points are observed, as expected for a two-level categorical variable. Checking for outliers, we find 1,113 points outside the 2-sigma band. That is 6.06% of the total 18,360, acceptable even if mildly elevated from the 4.55% that would be expected in a normal distribution.

The Cox regressions with and without stratification are, to a first approximation, consistent with proportional hazards for stays <200 days. The fact that the hazard ratio is nearly identical between the two choices suggests that the LOS change (evident also in Figs 5 and 6) is not caused by the change in dog characteristics noted in Table 5. This supports the comparisons in Fig 4 that tested consecutive periods without stratification. Dog size and age have been found to impact LOS [11,21,23–25], but stratification by size and age leaves the hazard ratio unchanged. In effect, the period effect appears superimposed on size and age effects.

The observed period variation may be the result of other dog covariates, socioeconomic community changes, or operational changes. For OC, an important operational change is known. OC retained some COVID-19-era restrictions into 2022–2024, particularly in visitor access and hours. When restrictions were gradually relaxed for a portion of the operating hours higher adoption rates were observed [48,49]. It is plausible that restrictions impacted outcomes other than adoptions, such as return-to-owner and transfer to rescue, which also involve visitor access, but this has not been studied. Industry recommendations for reducing LOS [8,33] emphasize visitor-friendly access and schedule, and OC restored adoption hours and visitor access to kennels in 2025 [50]. Given this background, shelter practices may have had a substantial effect on the LOS increase in OC.

A Cox ratio <1 corresponds to slower outcomes (longer LOS), but it is a ratio of hazards (instantaneous outcome rates), not a ratio of LOS. To quantify the consequences of the LOS change, we use the (non-stratified) metrics in Table 6. The mean ExitLOS of 20.8 with 387 monthly intakes post-COVID-19 translates to an expected resident count of 268. Had ExitLOS been maintained at 15.1 (the pre-COVID-19 mean), the resident count would instead be 195. A report [51] calculates that an attendant can care for 18–20 animals. The increase of 73 in resident count translates to 3–4 extra attendants every day (which requires about 7 full-time positions) for adequate care. LOS directly impacts resident count, which in turn impacts resource needs and quality of care – in addition to the fact that long stays are stressful to the animals [1,2].

Regardless of its origin, the presence of a significant LOS change shows the importance of monitoring LOS by period, so that changes can be detected. Here, we chose to compare two long periods, so that we can safely stratify by size and age and test the proportional hazards assumption. In practice, changes are noticeable even in shorter periods (Figs 4-5).

## 4 Discussion

This is a methodological proposal illustrated by empirical data. By using time-to-event statistical techniques, the ExitLOS computation allows the quantification and visualization of the evolution of LOS. A shelter can use it for early detection of LOS longitudinal shifts, inter-shelter benchmarking, or intra-shelter comparison of subsets of animals. To compute ExitLOS, a shelter only needs its tabulated intake and outcome data, simple computations to define calendar periods of interest, and statistical software with time-to-event capabilities including left-truncation and right-censoring. A more desirable option is for shelter databases to incorporate these computations in their LOS reporting. ExitLOS computations involve no delays. A monthly ExitLOS can be computed on the first day of the next month from readily available shelter data, allowing near-real-time assessments of shelter operations and their impact on capacity for care.

If LOS did not vary over time, then a shelter could study LOS for a fixed intake period far enough in the past, so that all animals have outcomes prior to the time of the study. For OC, we demonstrated that the LOS distribution does change with time, and a fixed past intake cohort does not correctly measure current conditions (or any specific past period’s conditions). LOS changes are significant, persist after accounting for animal size and age, and coincide with shelter operational changes. This shows that shelters can benefit from on-going LOS analysis.

Our two-month comparison shows how an outflux of animals from a shelter is misread as an LOS increase within the traditional (HistLOS) computation common in shelters. The reverse phenomenon, much more concerning, is when long-residents accumulate undetected by HistLOS computations. Shelters may, of course, read other indicators (e.g., the resident count) and adjust their LOS interpretation, but they are better served if they instead compute ExitLOS metrics avoiding the distortion.

In the limit where the study periods are much longer than most animals’ stay, HistLOS and ExitLOS converge, because as the period grows more observations are non-truncated and non-censored. But longer periods mean lower resolution and longer delay in recognizing LOS changes. Shorter periods allow LOS changes to be detected sooner, so that a shelter can assess external trends and the impact of internal operational decisions in a timely fashion and adapt accordingly.

The robustness of ExitLOS depends on the validity of its underlying statistical assumptions. The KM fits assume that truncation and censorship are non-informative, a reasonable approximation if period boundaries are based on a calendar schedule. The Cox proportional hazards regression further assumes that the effect of the periods (or of any other variables included in the regression) is proportional on the exit (outcome) rates. This is not always simple to ascertain. A starting point is to verify that the underlying KM curves do not cross in the central time interval of interest. As a practical matter, KM curves may be hard to distinguish in the first few days (which may be subject to mandatory holding periods) or in the extra-long stays of outliers.

A corollary of the examples presented here is that ExitLOS allows the comparison of shelters to each other within fixed calendar periods. The use of HistLOS for such comparisons has the same problems as its use for longitudinal comparisons illustrated in Figs 2–3.

In comparisons of periods or shelters, differences in animal demographics may influence the results. We stratified by size and age because our objective was to compare calendar periods rather than model animal-level LOS determinants. Maintaining the size and age variables constant over a dog’s stay in the shelter is a reasonable simplification. (Recall also that we rounded ages to years, and most dogs exit the shelter in a fraction of a year.) Nevertheless, refinement by size, age, and other categories (if sufficient data points are available) would allow a shelter to compare LOS in specific categories (over calendar periods or relative to its peers). LOS model refinement may also explore time-varying Cox regressions [52] and parametric distributions such as accelerated failure time models [53].

Pathway planning for newly arrived animals may rely on LOS expected at intake. But re-assessment and re-planning for resident animals requires estimation of a resident animal’s remaining LOS conditional on elapsed LOS (the animal’s stay so far). Modeling the full LOS distribution is necessary to obtain remaining LOS as a function of elapsed LOS. A dependency exists because LOS does not follow a geometric distribution. This is visually apparent in the KM plots of Fig 6. Furthermore, in the quarterly comparison the ratio of 90^th^ percentile to median ranges from 3.7 to 9.2, always higher than the asymptotic 3.3 of a geometric distribution.

Rigorous retrospective cohort studies [21,23–26] using KM or Cox take a static view of LOS because they are not concerned with changes in a shelter’s LOS from quarter to quarter or year to year. Animal shelters themselves have a dynamic view, primarily interested in longitudinal LOS changes, but their static approach (HistLOS) produces distorted metrics. Many shelters do not reference LOS in their statistics at all. The organizations that compute and track LOS are clearly attentive to tracking their metrics and improving their performance, but they need better tools.

The OC Strategic Plan [54] includes LOS targets of 8 days for fast-track and 15 days for slow-track dogs. In a public report, OC stated that average LOS has skyrocketed across the nation but remains at 11 days in OC [55], without specifying calendar period, method of averaging, or animal species. After press reports cited a 60% increase in LOS of adult dogs [56], another OC document [57] provided no clear LOS metrics. OC had no relevant computation of length of stay statistics when queried [58]. In this instance, then, changes in length of stay appear to have been missed and this may have contributed to staffing shortfalls and overshooting of capacity for care [51].

In conclusion, ExitLOS rigorously accounts for animals whose stay overlaps with the start or the end a calendar period of interest, by deploying left-truncation and right-censoring in KM and Cox regressions. Thus, ExitLOS addresses the discrepancy inherent in existing shelter LOS computations. For OC, ExitLOS KM curves show statistically significant variation over quarterly periods (and monthly, when a sharp change occurs). For longer periods, the variation exists even after we account for the mix of incoming dog sizes and ages and is therefore informative about shelter operations. ExitLOS with additional stratification or covariates may alter how shelters evaluate their operations, by enabling valid comparisons across periods, peer shelters, or animal sub-populations. As shelters expand and refine their use of this metric, future research can build on these methods to address evolving operational needs.

## Supporting information

S1 Fig Schoenfeld residuals for the stratified Cox proportional hazards regression of the pre-COVID-19 and post-COVID-19 periods. Stratification is by dog size and age. The pre-COVID-19 period is July 1, 2018 – February 29, 2020; the post-COVID-19 period is January 1, 2022 – October 31, 2023. The red line (with dashed lines for 95% CI) is a smoothed average of the residuals.
(TIFF)

S2 Fig Deviance residuals for the stratified Cox proportional hazards regression of the pre-COVID-19 and post-COVID-19 periods. Stratification is by dog size and age. The pre-COVID-19 period is July 1, 2018 – February 29, 2020; the post-COVID-19 period is January 1, 2022 – October 31, 2023. There are 1,113 (6.06%) of 18,360 of points outside the 2-sigma band, slightly more than the expected 4.55%.
(TIFF)
