# Supplementary figures and images for: Use of Kaplan-Meier and Cox regressions in the distribution of length of stay in animal shelters for pre-specified calendar periods: Definition, computation, and examples of dog length of stay in orange county California
Source: PLoS One. 2026 Jan 30;21(1):e0342102. doi: 10.1371/journal.pone.0342102 (PMC12857969; doi:10.1371/journal.pone.0342102)

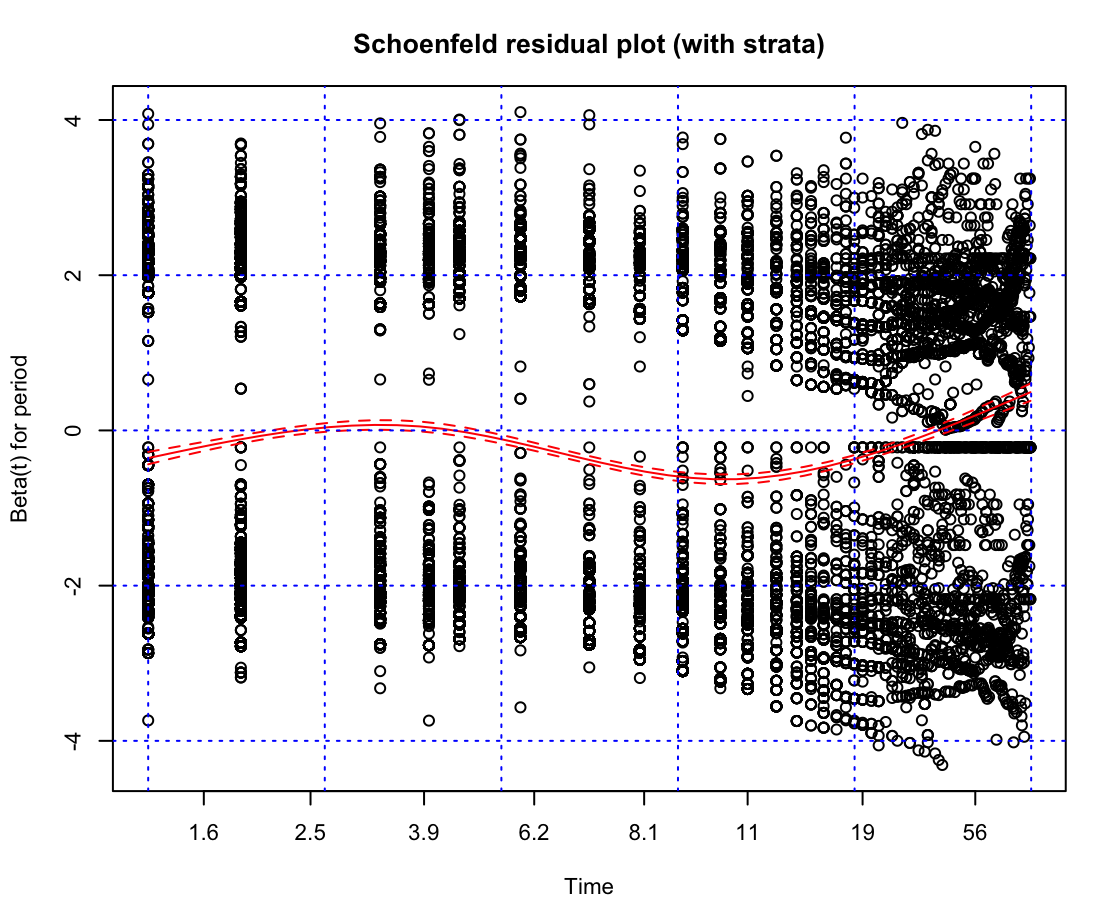

Supplement: S1 Fig — Stratification is by dog size and age. The pre-COVID-19 period is July 1, 2018 – February 29, 2020; the post-COVID-19 period is January 1, 2022 – October 31, 2023. The red line (with dashed lines for 95% CI) is a smoothed average of the residuals. (TIFF) [file pone.0342102.s001.tiff]

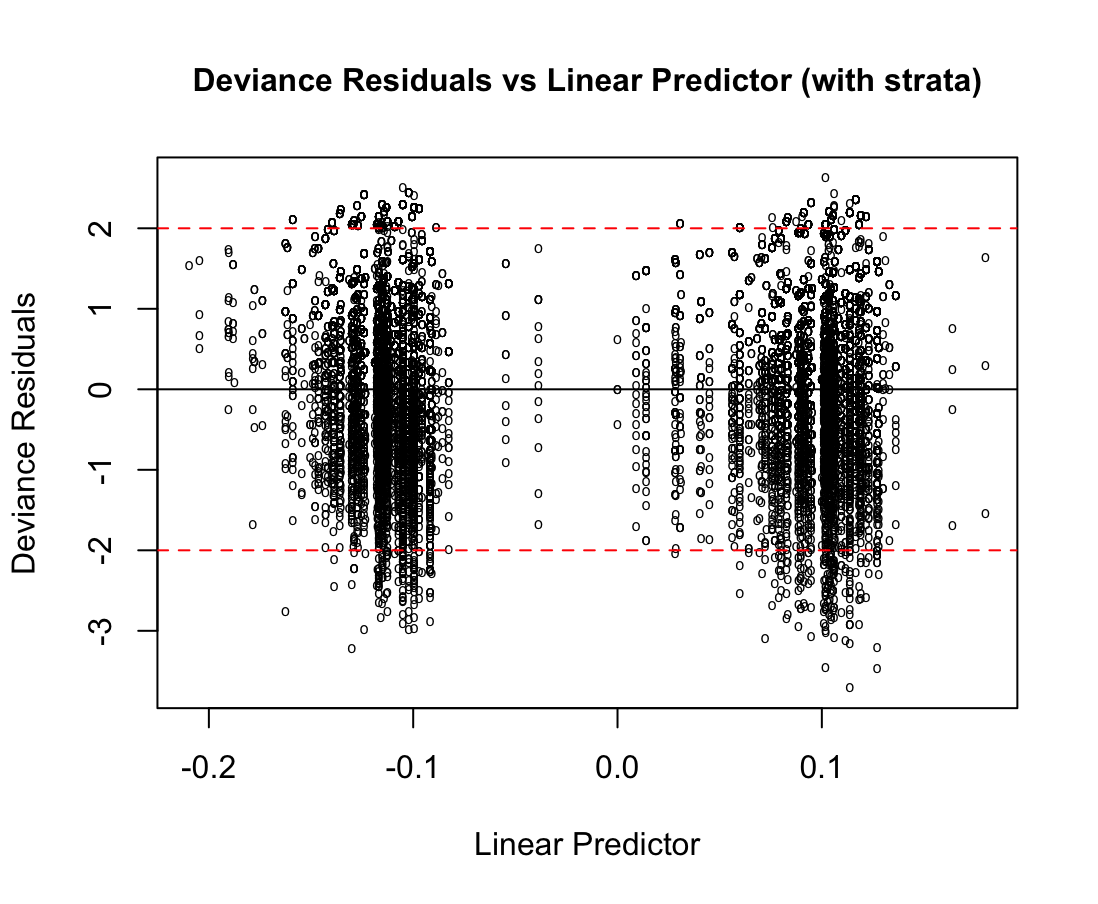

Supplement: S2 Fig — Stratification is by dog size and age. The pre-COVID-19 period is July 1, 2018 – February 29, 2020; the post-COVID-19 period is January 1, 2022 – October 31, 2023. There are 1,113 (6.06%) of 18,360 of points outside the 2-sigma band, slightly more than the expected 4.55%. (TIFF) [file pone.0342102.s002.tiff]
